# (Ethyl­enediamine-κ^2^ *N*,*N*′)bis­(perchlorato-κ*O*)bis­(pyridine-κ*N*)copper(II)

**DOI:** 10.1107/S1600536812029868

**Published:** 2012-07-07

**Authors:** Ali Ourari, Nawel Bounab, Sofiane Bouacida, Djouhra Aggoun

**Affiliations:** a Laboratoire d’Electrochimie, d’Ingénierie Moléculaire et de Catalyse Redox (LEIMCR), Faculté des Sciences de l’Ingénieur, Université Farhat Abbas, Sétif 19000, Algeria; b Unité de Recherche de Chimie de l’Environnement et Moléculaire Structurale (CHEMS), Université Mentouri-Constantine, 25000, Algeria

## Abstract

In the title compound, [Cu(ClO_4_)_2_(C_2_H_8_N_2_)(C_5_H_5_N)_2_], the Cu^II^ cation is located on a twofold rotation axis and is coordinated by four N and two O atoms in a tetragonally distorted octahedral geometry. The crystal packing can be described as ClO_4_ tetra­hedra and CuN_4_O_2_ octa­hedra alternating in a zigzag fashion along the *c* axis. The structure is stabilized by intermolecular N—H⋯O and C—H⋯O hydrogen bonds, as well as π–π interactions [centroid–centroid distance = 3.7179 (15) Å].

## Related literature

For synthesis and applications of similar compounds, see: De Stefano *et al.* (1999 ▶); Sing *et al.* (2004 ▶); Elliot & Herchenhart (1982 ▶); Moncol *et al.* (2008 ▶); Costes *et al.* (1998 ▶).
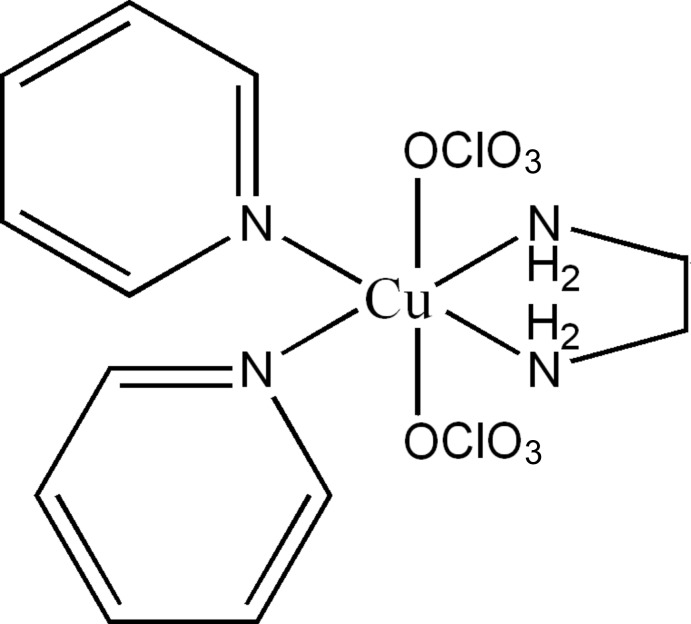


## Experimental

### 

#### Crystal data

[Cu(ClO_4_)_2_(C_2_H_8_N_2_)(C_5_H_5_N)_2_]
*M* *_r_* = 480.74
Monoclinic, 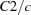

*a* = 7.697 (1) Å
*b* = 17.238 (2) Å
*c* = 14.206 (1) Å
β = 100.551 (1)°
*V* = 1853.0 (3) Å^3^
*Z* = 4
Mo *K*α radiation
μ = 1.52 mm^−1^
*T* = 295 K
0.17 × 0.15 × 0.13 mm

#### Data collection

Nonius KappaCCD diffractometer
4657 measured reflections
2400 independent reflections
2117 reflections with *I* > 2σ(*I*)
*R*_int_ = 0.026

#### Refinement

*R*[*F* ^2^ > 2σ(*F* ^2^)] = 0.047
*wR*(*F* ^2^) = 0.136
*S* = 1.08
2400 reflections
124 parameters
H-atom parameters constrained
Δρ_max_ = 0.87 e Å^−3^
Δρ_min_ = −0.75 e Å^−3^

### 

Data collection: *COLLECT* (Nonius, 2004 ▶); cell refinement: *SCALEPACK* (Otwinowski & Minor, 1997 ▶); data reduction: *DENZO* (Otwinowski & Minor, 1997 ▶) and *SCALEPACK*; program(s) used to solve structure: *SIR2002* (Burla *et al.*, 2005 ▶); program(s) used to refine structure: *SHELXL97* (Sheldrick, 2008 ▶); molecular graphics: *ORTEP-3 for Windows* (Farrugia, 1997 ▶) and *DIAMOND* (Brandenburg & Berndt, 2001 ▶); software used to prepare material for publication: *WinGX* (Farrugia, 1999 ▶).

## Supplementary Material

Crystal structure: contains datablock(s) global, I. DOI: 10.1107/S1600536812029868/zj2084sup1.cif

Structure factors: contains datablock(s) I. DOI: 10.1107/S1600536812029868/zj2084Isup2.hkl

Additional supplementary materials:  crystallographic information; 3D view; checkCIF report

## Figures and Tables

**Table 1:** Selected bond lengths (Å)

N1—Cu1	2.017 (2)
N2—Cu1	2.0206 (19)
O11—Cu1	2.613 (3)

**Table 2:** Hydrogen-bond geometry (Å, °)

*D*—H⋯*A*	*D*—H	H⋯*A*	*D*⋯*A*	*D*—H⋯*A*
N1—H1*C*⋯O12	0.90	2.44	3.213 (3)	144
N1—H1*C*⋯O12^i^	0.90	2.50	3.247 (3)	140
N1—H1*D*⋯O14^ii^	0.90	2.23	3.111 (4)	166
C2—H2⋯O11	0.93	2.53	3.076 (4)	118
C5—H5⋯O13^iii^	0.93	2.57	3.208 (4)	126

Symmetry codes: (i) 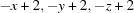; (ii) 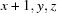; (iii) 
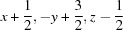
.

**Fig. 1.**
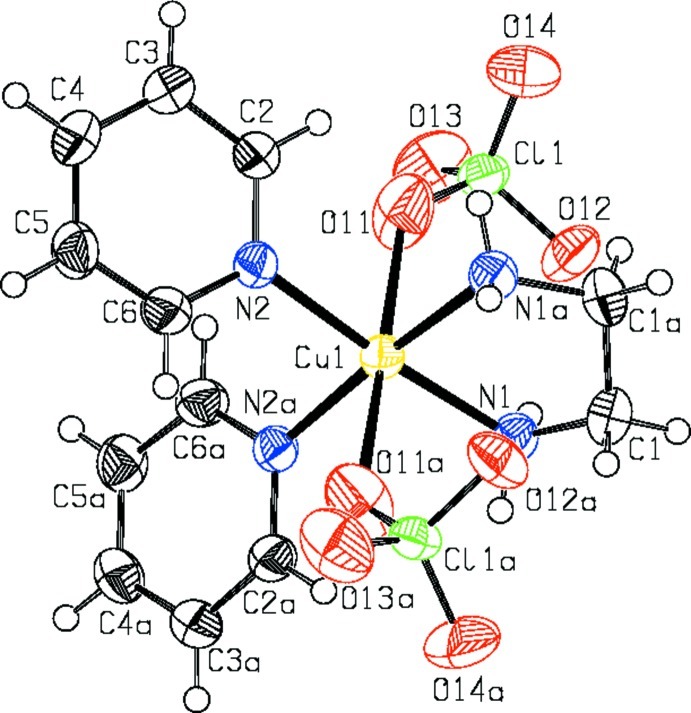
Drawing the molecular geometry of (I) with the atom-labelling scheme. Displacement ellipsoids are drawn at the 50% probability level. H atoms are represented as small spheres of arbitrary radii.

**Fig. 2.**
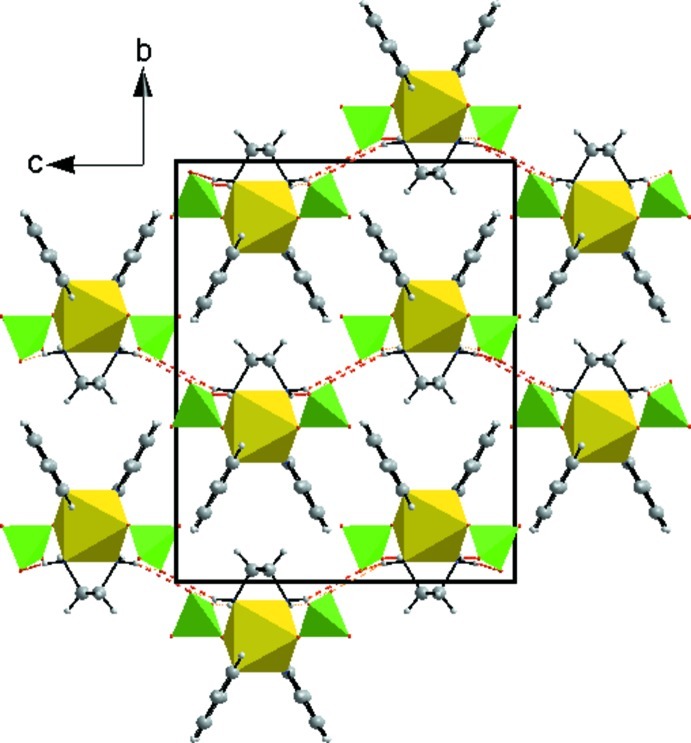
Alternating polyhedra of (I) viewed viaa axis showing ClO4 tetrahedra in green and CuN4O2 octahedra in yellow. Hydrogen bond [N—H···O] are in (red) dashed line.

## supplementary crystallographic information

### Comment

Aliphatic and aromatic amines such as ethylenediamine and pyridine are commonly known as the good chelating properties towards transition metals (De Stefano *et al.*, 1999). In this case, the title compound was obtained when mixing ethylenediamine, 2-hydroxy-6-[3-(1*H*-pyrrol-1-yl)propoxy]acetophenone and perchlorate of copper in a methanolic solution. This work have been focused the synthesis of half-units for the preparation of non-symmetrical Schiff base ligands (Costes *et al.*, 1998) but, the resulting compound was not the expected material. Thus, it seems that the preferential formation of unexpected copper complex is probably due to the no heating of the mixture at the first moments after mixing the different reagents or to the higher affinity the copper ion towards the amines previously indicated (Moncol *et al.*, 2008). This is in accordance with high donor effect of these both amines as reported in the literature for the synthesis of coordination compounds using aliphatic (Sing *et al.*, 2004)) and aromatic amines such as bipyridinc ligands (Elliot & Herchenhart, 1982). We report here the synthesis of title compound and its crystal structure. The molecular geometry of structure, (I), and the atomic numbering used, is illustrated in Fig. 1. The Cu^II^ ion is coordinated in an irregular octahedral geometry by four N atoms *via* two pyridine and one ethylenediamine moiety and two O atoms *via* two perchlorate. The bond lengths for coordination Cu^II^ sphere is ranging from 2.017 (2) to 2.0206 (19) Å for Cu—N distances and is 2.613 (3) Å for Cu—O distance (Table 1). The crystal packing in the title structure can be described by alterning ClO_4_ tetrahedra and CuN_4_O_2_ octahedra of complex in zigzag along the *c* axis (Fig. 2). It's stabilized by intermolecular N—H···O hydrogen bonding (Table 2) and π–π interactions.

### Experimental

259 mg (1 mmol) of 2-hydroxy-6-[3-(1*H*-pyrrol-1-yl)propoxy]acetophenone, 373 mg (1 mmol) of copper perchlorate hexahydrated and an excess of pyridine were dissolved in 12 ml of methanol. This solution was placed in a three necked flask surmounted by a condenser before to add it dropwisely a methanolic solution (8 ml) containing 60 mg (1 mmol) of ethylenediamine. This mixture was kept under nitrogen atmosphere and stirring for about 2 h to observe an abundant mallow precipitate. This solid was recovered by filtration, copiously washed with methanol and the suitable crystals were obtained by slow evaporation from the filtrate.

### Refinement

The remaining H atoms were localized on Fourier maps but introduced in calculated positions and treated as riding on their parent atoms (C and N) with C—H = 0.97 Å (methylene) or 0.93 Å (aromatic) and N—H = 0.90 Å with *U*_iso_(H) = 1.2*U*_eq_(C or N).

### Crystal data

**Table:**

[Cu(ClO_4_)_2_(C_2_H_8_N_2_)(C_5_H_5_N)_2_]	*F*(000) = 980
*M**_r_* = 480.74	*D*_x_ = 1.723 Mg m^−^^3^
Monoclinic, *C*2/*c*	Mo *K*α radiation, λ = 0.71073 Å
*a* = 7.697 (1) Å	Cell parameters from 2471 reflections
*b* = 17.238 (2) Å	θ = 1.0–28.7°
*c* = 14.206 (1) Å	µ = 1.52 mm^−^^1^
β = 100.551 (1)°	*T* = 295 K
*V* = 1853.0 (3) Å^3^	Prism, colourless
*Z* = 4	0.17 × 0.15 × 0.13 mm

### Data collection

**Table:**

Nonius KappaCCD diffractometer	2117 reflections with *I* > 2σ(*I*)
Radiation source: Enraf–Nonius FR590	*R*_int_ = 0.026
Graphite monochromator	θ_max_ = 28.7°, θ_min_ = 2.9°
Detector resolution: 9 pixels mm^-1^	*h* = −10→10
CCD rotation images, thick slices scans	*k* = −21→23
4657 measured reflections	*l* = −19→19
2400 independent reflections	

### Refinement

**Table:**

Refinement on *F*^2^	Primary atom site location: structure-invariant direct methods
Least-squares matrix: full	Secondary atom site location: difference Fourier map
*R*[*F*^2^ > 2σ(*F*^2^)] = 0.047	Hydrogen site location: inferred from neighbouring sites
*wR*(*F*^2^) = 0.136	H-atom parameters constrained
*S* = 1.08	*w* = 1/[σ^2^(*F*_o_^2^) + (0.0907*P*)^2^ + 1.252*P*] where *P* = (*F*_o_^2^ + 2*F*_c_^2^)/3
2400 reflections	(Δ/σ)_max_ < 0.001
124 parameters	Δρ_max_ = 0.87 e Å^−^^3^
0 restraints	Δρ_min_ = −0.75 e Å^−^^3^

### Special details

**Table:**

Geometry. All e.s.d.'s (except the e.s.d. in the dihedral angle between two l.s. planes) are estimated using the full covariance matrix. The cell e.s.d.'s are taken into account individually in the estimation of e.s.d.'s in distances, angles and torsion angles; correlations between e.s.d.'s in cell parameters are only used when they are defined by crystal symmetry. An approximate (isotropic) treatment of cell e.s.d.'s is used for estimating e.s.d.'s involving l.s. planes.
Refinement. Refinement of *F*^2^ against ALL reflections. The weighted *R*-factor *wR* and goodness of fit *S* are based on *F*^2^, conventional *R*-factors *R* are based on *F*, with *F* set to zero for negative *F*^2^. The threshold expression of *F*^2^ > σ(*F*^2^) is used only for calculating *R*-factors(gt) *etc*. and is not relevant to the choice of reflections for refinement. *R*-factors based on *F*^2^ are statistically about twice as large as those based on *F*, and *R*-factors based on ALL data will be even larger.

### Fractional atomic coordinates and isotropic or equivalent isotropic displacement parameters (Å^2^)

**Table:**

	*x*	*y*	*z*	*U*_iso_*/*U*_eq_	
C1	1.0960 (4)	1.02744 (14)	0.77477 (19)	0.0420 (5)	
H1A	1.1727	1.0316	0.7279	0.050*	
H1B	1.1191	1.0711	0.8183	0.050*	
C2	0.6763 (3)	0.78272 (14)	0.66706 (18)	0.0373 (5)	
H2	0.6232	0.8232	0.6946	0.045*	
C3	0.5711 (3)	0.72425 (15)	0.6214 (2)	0.0434 (5)	
H3	0.4492	0.7256	0.6176	0.052*	
C4	0.6494 (4)	0.66365 (15)	0.5815 (2)	0.0443 (6)	
H4	0.5815	0.6230	0.5514	0.053*	
C5	0.8304 (4)	0.66431 (15)	0.5869 (2)	0.0453 (6)	
H5	0.8862	0.6242	0.5603	0.054*	
C6	0.9274 (3)	0.72536 (15)	0.63244 (18)	0.0413 (5)	
H6	1.0489	0.7261	0.6348	0.050*	
N1	1.1296 (3)	0.95373 (12)	0.82833 (15)	0.0375 (4)	
H1C	1.0923	0.9576	0.8846	0.045*	
H1D	1.2463	0.9437	0.8405	0.045*	
N2	0.8526 (3)	0.78360 (11)	0.67335 (14)	0.0331 (4)	
O11	0.7648 (4)	0.86617 (14)	0.8613 (2)	0.0731 (8)	
O12	0.8304 (3)	0.97537 (13)	0.95701 (16)	0.0552 (5)	
O13	0.6922 (4)	0.86854 (17)	1.0120 (2)	0.0783 (9)	
O14	0.5379 (3)	0.9459 (2)	0.8925 (3)	0.0996 (11)	
Cl1	0.70478 (7)	0.91475 (3)	0.93029 (4)	0.0372 (2)	
Cu1	1.0000	0.86690 (2)	0.7500	0.03224 (18)	

### Atomic displacement parameters (Å^2^)

**Table:**

	*U*^11^	*U*^22^	*U*^33^	*U*^12^	*U*^13^	*U*^23^
C1	0.0521 (15)	0.0313 (11)	0.0429 (12)	−0.0063 (10)	0.0091 (11)	−0.0030 (9)
C2	0.0338 (11)	0.0349 (11)	0.0425 (12)	0.0019 (8)	0.0053 (9)	−0.0036 (9)
C3	0.0361 (12)	0.0412 (13)	0.0515 (14)	−0.0038 (9)	0.0043 (10)	−0.0029 (10)
C4	0.0472 (14)	0.0382 (13)	0.0462 (13)	−0.0093 (10)	0.0048 (11)	−0.0065 (10)
C5	0.0515 (15)	0.0365 (12)	0.0500 (14)	0.0010 (10)	0.0154 (11)	−0.0102 (10)
C6	0.0352 (12)	0.0423 (13)	0.0469 (13)	0.0015 (9)	0.0089 (10)	−0.0059 (10)
N1	0.0377 (10)	0.0357 (10)	0.0373 (9)	−0.0011 (8)	0.0022 (8)	−0.0035 (8)
N2	0.0334 (9)	0.0302 (9)	0.0347 (9)	−0.0003 (7)	0.0031 (7)	−0.0025 (7)
O11	0.0894 (19)	0.0654 (16)	0.0759 (16)	−0.0184 (12)	0.0450 (15)	−0.0287 (12)
O12	0.0490 (11)	0.0538 (12)	0.0612 (12)	−0.0194 (9)	0.0058 (9)	−0.0112 (10)
O13	0.085 (2)	0.091 (2)	0.0637 (16)	−0.0214 (14)	0.0274 (14)	0.0171 (13)
O14	0.0421 (13)	0.085 (2)	0.154 (3)	0.0104 (13)	−0.0286 (16)	−0.007 (2)
Cl1	0.0288 (3)	0.0418 (3)	0.0406 (3)	−0.00471 (19)	0.0051 (2)	−0.0045 (2)
Cu1	0.0302 (3)	0.0291 (3)	0.0357 (3)	0.000	0.00142 (16)	0.000

### Geometric parameters (Å, º)

**Table:**

C1—N1	1.479 (3)	C6—N2	1.342 (3)
C1—C1^i^	1.517 (5)	C6—H6	0.9300
C1—H1A	0.9700	N1—H1C	0.9000
C1—H1B	0.9700	N1—H1D	0.9000
C2—N2	1.343 (3)	N1—Cu1	2.017 (2)
C2—C3	1.379 (3)	N2—Cu1	2.0206 (19)
C2—H2	0.9300	O11—Cu1	2.613 (3)
C3—C4	1.379 (4)	O11—Cl1	1.428 (2)
C3—H3	0.9300	O12—Cl1	1.427 (2)
C4—C5	1.381 (4)	O13—Cl1	1.425 (3)
C4—H4	0.9300	O14—Cl1	1.406 (2)
C5—C6	1.381 (4)	Cu1—N1^i^	2.017 (2)
C5—H5	0.9300	Cu1—N2^i^	2.0206 (19)
			
N1—C1—C1^i^	107.61 (17)	C1—N1—H1D	109.8
N1—C1—H1A	110.2	Cu1—N1—H1D	109.8
C1^i^—C1—H1A	110.2	H1C—N1—H1D	108.3
N1—C1—H1B	110.2	C6—N2—C2	118.1 (2)
C1^i^—C1—H1B	110.2	C6—N2—Cu1	121.47 (17)
H1A—C1—H1B	108.5	C2—N2—Cu1	120.30 (15)
N2—C2—C3	122.6 (2)	Cl1—O11—Cu1	139.39 (15)
N2—C2—H2	118.7	O14—Cl1—O13	109.3 (2)
C3—C2—H2	118.7	O14—Cl1—O12	110.42 (17)
C2—C3—C4	118.9 (2)	O13—Cl1—O12	109.67 (17)
C2—C3—H3	120.5	O14—Cl1—O11	110.5 (2)
C4—C3—H3	120.5	O13—Cl1—O11	108.03 (18)
C3—C4—C5	118.9 (2)	O12—Cl1—O11	108.87 (15)
C3—C4—H4	120.5	N1—Cu1—N1^i^	84.17 (12)
C5—C4—H4	120.5	N1—Cu1—N2^i^	93.31 (9)
C6—C5—C4	119.1 (2)	N1^i^—Cu1—N2^i^	175.59 (8)
C6—C5—H5	120.5	N1—Cu1—N2	175.59 (8)
C4—C5—H5	120.5	N1^i^—Cu1—N2	93.31 (9)
N2—C6—C5	122.4 (2)	N2^i^—Cu1—N2	89.42 (11)
N2—C6—H6	118.8	N1—Cu1—O11	89.85 (8)
C5—C6—H6	118.8	N1^i^—Cu1—O11	90.56 (10)
C1—N1—Cu1	109.36 (15)	N2^i^—Cu1—O11	93.06 (9)
C1—N1—H1C	109.8	N2—Cu1—O11	86.55 (8)
Cu1—N1—H1C	109.8		
			
N2—C2—C3—C4	0.8 (4)	C1—N1—Cu1—N2	−69.6 (10)
C2—C3—C4—C5	−1.3 (4)	C1—N1—Cu1—O11	−104.80 (17)
C3—C4—C5—C6	0.3 (4)	C6—N2—Cu1—N1	−179 (100)
C4—C5—C6—N2	1.3 (4)	C2—N2—Cu1—N1	−3.5 (11)
C1^i^—C1—N1—Cu1	39.1 (3)	C6—N2—Cu1—N1^i^	125.50 (19)
C5—C6—N2—C2	−1.8 (4)	C2—N2—Cu1—N1^i^	−58.60 (19)
C5—C6—N2—Cu1	174.2 (2)	C6—N2—Cu1—N2^i^	−51.04 (17)
C3—C2—N2—C6	0.7 (4)	C2—N2—Cu1—N2^i^	124.9 (2)
C3—C2—N2—Cu1	−175.34 (19)	C6—N2—Cu1—O11	−144.1 (2)
Cu1—O11—Cl1—O14	107.9 (3)	C2—N2—Cu1—O11	31.77 (19)
Cu1—O11—Cl1—O13	−132.5 (3)	Cl1—O11—Cu1—N1	17.1 (3)
Cu1—O11—Cl1—O12	−13.5 (4)	Cl1—O11—Cu1—N1^i^	−67.1 (3)
C1—N1—Cu1—N1^i^	−14.22 (12)	Cl1—O11—Cu1—N2^i^	110.4 (3)
C1—N1—Cu1—N2^i^	162.14 (16)	Cl1—O11—Cu1—N2	−160.4 (3)

Symmetry code: (i) −*x*+2, *y*, −*z*+3/2.

### Hydrogen-bond geometry (Å, º)

**Table:**

*D*—H···*A*	*D*—H	H···*A*	*D*···*A*	*D*—H···*A*
N1—H1*C*···O12	0.90	2.44	3.213 (3)	144
N1—H1*C*···O12^ii^	0.90	2.50	3.247 (3)	140
N1—H1*D*···O14^iii^	0.90	2.23	3.111 (4)	166
C2—H2···O11	0.93	2.53	3.076 (4)	118
C5—H5···O13^iv^	0.93	2.57	3.208 (4)	126

Symmetry codes: (ii) −*x*+2, −*y*+2, −*z*+2; (iii) *x*+1, *y*, *z*; (iv) *x*+1/2, −*y*+3/2, *z*−1/2.
